# NRF2 in kidney physiology and disease

**DOI:** 10.14814/phy2.15961

**Published:** 2024-02-28

**Authors:** Corry D. Bondi, Hannah L. Hartman, Roderick J. Tan

**Affiliations:** ^1^ Renal‐Electrolyte Division, Department of Medicine University of Pittsburgh School of Medicine Pittsburgh Pennsylvania USA

**Keywords:** bardoxolone methyl, chronic kidney disease, electrophiles, oxidative stress, proteinuria

## Abstract

The role of NRF2 in kidney biology has received considerable interest over the past decade. NRF2 transcriptionally controls genes responsible for cellular protection against oxidative and electrophilic stress and has anti‐inflammatory functions. NRF2 is expressed throughout the kidney and plays a role in salt and water handling. In disease, animal studies show that NRF2 protects against tubulointerstitial damage and reduces interstitial fibrosis and tubular atrophy, and may slow progression of polycystic kidney disease. However, the role of NRF2 in proteinuric glomerular diseases is controversial. Although the NRF2 inducer, bardoxolone methyl (CDDO‐Me), increases glomerular filtration rate in humans, it has not been shown to slow disease progression in diabetic kidney disease and Alport syndrome. Furthermore, bardoxolone methyl was associated with negative effects on fluid retention, proteinuria, and blood pressure. Several animal studies replicate findings of worsened proteinuria and a more rapid progression of kidney disease, although considerable controversy exists. It is clear that further study is needed to better understand the effects of NRF2 in the kidney. This review summarizes the available data to clarify the promise and risks associated with targeting NRF2 activity in the kidney.

## 1 INTRODUCTION

The nuclear factor erythroid 2‐related factor 2 (NRF2) system is an important mechanism for mitigating cellular stress. NRF2 upregulates genes that alleviate oxidative stress and detoxify electrophilic compounds that threaten cellular homeostasis (Yamamoto et al., 2018). NRF2 also plays an important role in reducing inflammation. Not surprisingly, NRF2 has been implicated in a variety of human diseases, including heart, lung, liver, and kidney diseases as well as in cancer biology (Yamamoto et al., 2018).

**FIGURE 1:**
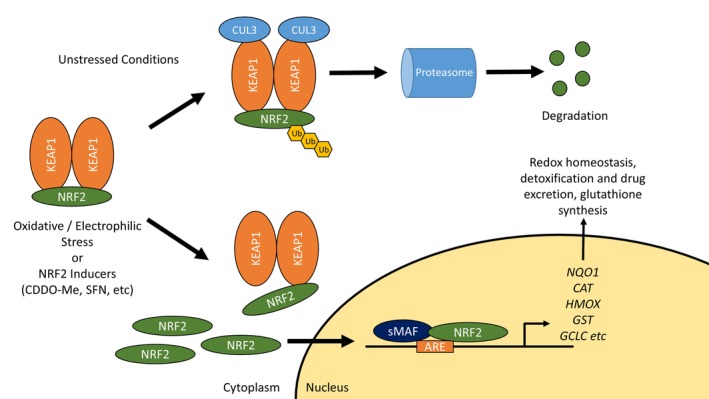
The KEAP1/NRF2 Pathway. KEAP1 is the endogenous inhibitor of NRF2, directing it for ubiquitination and subsequent degradation via the proteasome. However, in the presence of oxidative stress, electrophiles, or NRF2‐inducing agents, KEAP1 is modified and NRF2 is allowed to accumulate. Free NRF2 translocates to the nucleus and partners with sMAF transcription factors to upregulate gene expression. Many of these genes have antioxidant, detoxifying, or other cytoprotective properties. KEAP1, Kelch‐like associated protein 1; NRF2, nuclear factor 2 erythroid 2; sMAF, small musculo‐aponeurotic fibrosarcoma; ARE, antioxidant response element; *NQO1*, NAD(P)H quinone dehydrogenase 1; *CAT*, catalase; *HMOX*, heme oxygenase 1; *GST*, glutathione S‐transferases; *GCLC*, glutamate‐cysteine ligase catalytic subunit.

NRF2 is a member of the NF‐E2 family of Cap'n'Collar basic leucine zipper DNA‐binding transcription factors (Moi et al., 1994). NRF2 is restrained by its endogenous inhibitor Kelch‐like ECH‐associated protein 1 (KEAP1). Under normal conditions, KEAP1 ubiquitinates NRF2 and directs it for proteasomal degradation (Cullinan et al., 2004; Kobayashi et al., 2004; Nguyen et al., 2003; Zhang et al., 2004). These two proteins work in concert, with KEAP1 serving as a sensor for reactive oxygen species and electrophiles (Nezu, Suzuki, & Yamamoto, 2017). These modify thiol side chains of key cysteine residues on KEAP1, disrupting its interaction with NRF2 or its ability to degrade it (Yamamoto et al., 2018). NRF2 accumulates, translocates to the nucleus, and forms a heterodimer with small musculo‐aponeurotic fibrosarcoma (sMAF) transcription factors (Itoh et al., 1997). The sMAF‐NRF2 heterodimer binds to antioxidant and electrophile response elements to upregulate target genes including NAD(P)H quinone dehydrogenase 1 (*NQO1*), glutathione S‐transferases (*GSTs)*, catalase (*CAT*), and heme oxygenase 1 (*HMOX*) (Figure 1). These and other upregulated genes have roles in mitigating oxidative stress, participating in detoxification pathways, and increasing glutathione synthesis, among other effects (Nezu, Suzuki, & Yamamoto, 2017). In short, the activation of NRF2 by oxidative or electrophilic stress leads to the upregulation of cellular defenses against these insults.

NRF2 has anti‐inflammatory effects, including negative effects on NF‐κB and gene transcription of IL‐1β and IL‐6 (van der Horst et al., 2022). NRF2 also impacts metabolism, obesity, and insulin resistance (Chartoumpekis & Kensler, 2013). In aggregate, these functions indicate a critical role for NRF2 in health and disease.

A number of chemicals have been isolated or developed to induce NRF2. These include sulforaphane, dimethyl fumarate, and the triterpenoids bardoxolone methyl (CDDO‐Me) and bardoxolone imidazole (CDDO‐Im). These act by disrupting KEAP1‐mediated NRF2 degradation (Yamamoto et al., 2018). In addition, NRF2 is regulated by glycogen synthase kinase‐3β (GSK‐3β) and β‐TrCP, the phosphatidylinositol‐3‐kinase/AKT (PI3K/AKT) pathway, extracellular signal‐regulated kinase (ERK), C‐Jun N‐terminal kinase (JNK), and PKR‐like endoplasm reticulum kinase (PERK) (Guerrero‐Hue et al., 2020).

## 2 NRF2 EXPRESSION IN THE KIDNEY

According to the Human Protein Atlas, NRF2 has higher expression in renal tubules compared to glomeruli under normal conditions (Uhlen et al., 2015). However, our group and others have observed glomerular upregulation during disease (Jiang et al., 2010; Jiang et al., 2014; Rush et al., 2021). Although its protein abundance is informative, NRF2 activity may be more accurately reflected in the expression of target genes such as *NQO1*. In the Human Protein Atlas, NQO1 is found in both glomeruli and distal tubules (Uhlen et al., 2015).

In mice, NRF2 protein is present in proximal (Cuevas et al., 2015) and distal (Liu et al., 2019) tubules with some glomerular expression (Henique et al., 2016). Interestingly, NQO1 is constitutively expressed in the proximal tubule, but can be induced in the distal tubules via administration of NRF2 inducers or by genetic knockdown of KEAP1 (Jobbagy et al., 2020). Glomerular expression of NQO1 is enhanced during disease (Moon et al., 2020). Interestingly, severe injuries can maladaptively decrease NRF2 activity and *Nqo1* expression in renal tubules (Bondi et al., 2022).

## 3 THE ROLE OF NRF2 IN KIDNEY PHYSIOLOGY

Using a transcriptomic and proteomic approach, Shelton and colleagues determined that kidneys from *Nrf2* knockout mice are deficient in genes and proteins that regulate glutathione synthesis and conjugation, redox balance, metabolism and removal of xenobiotics, and generation of NAD(P)H (Shelton et al., 2015). Ingenuity Pathway Analysis similarly revealed roles in “xenobiotic metabolism signaling,” “aryl hydrocarbon receptor signaling,” “pregnane X receptor (PXR)/retinoid X receptor (RXR) activation,” and “glutathione‐mediated detoxification.” These findings confirm the known roles for NRF2 in antioxidant and detoxifying functions and also suggest an important role in regulating general kidney homeostasis (Shelton et al., 2015).

NRF2 activity appears to have a reversible pharmacodynamic effect on glomerular filtration rate (GFR). The NRF2 inducer, bardoxolone methyl, lowers serum creatinine in humans (Hong et al., 2012), an effect that ultimately led to its investigation for the treatment of chronic kidney disease (CKD). Multiple human trials have confirmed this effect, as well as reversal after drug washout (de Zeeuw et al., 2013; Pergola et al., 2011; Warady et al., 2022). This is a true effect on glomerular filtration, since bardoxolone methyl increases inulin clearance (Nangaku et al., 2020). The mechanism in humans is unknown, but in rodents NRF2 activity increases expression of the sodium‐glucose cotransporter 2 (SGLT2) (Zhao et al., 2021). Higher SGLT2 expression could increase GFR through effects on tubulo‐glomerular feedback (Vallon & Verma, 2021). Other experimental studies showed that NRF2 enhancement could increase glomerular surface area (and GFR) via effects on mesangial (Ding et al., 2013) or podocyte contractility (Kidokoro et al., 2023).

NRF2 affects renal salt and water handling. In elegant mouse studies, high NRF2 activity induced by genetic *Keap1* deletion reduced aquaporin‐2 expression, leading to nephrogenic diabetes insipidus (DI) and hydronephrosis (Noel et al., 2016; Suzuki et al., 2017). Meanwhile, mice with *Keap1* hypomorphism (reduced expression instead of a complete deletion) exhibited only mild hyposthenuria and normal urinary concentrating ability. The hypomorphic mice also had reduced expression of the Na^+^‐Cl^−^ cotransporter (NCC) and were surprisingly protected against lithium‐induced DI (Jobbagy et al., 2020). The puzzling difference in phenotype could be related to the graded increase in NRF2 activity caused by *Keap1* hypomorphism (high) compared to complete knockout (very high). This would be an example of hormesis (Bhakta‐Guha & Efferth, 2015), with a deleterious outcome being determined by the greater NRF2 activity in the knockout.

The effect of NRF2 on blood pressure appears to be complex and context‐dependent. Many studies demonstrate that NRF2 activity increases blood pressure. In humans, bardoxolone methyl increased or tended to increase blood pressure (de Zeeuw et al., 2013; Pergola et al., 2011). However, at least one study in mice showed that bardoxolone methyl can reduce blood pressure (Hisamichi et al., 2018). Our work using radiotelemetry found that *Keap1* hypomorphic mice exhibit higher blood pressure, specifically during the inactive daytime portion of their diurnal cycle (mice are nocturnal). Unlike wild‐type mice, these hypomorphic mice also had defects in blood pressure dipping during sleep when challenged with chronic angiotensin II infusion (Rush et al., 2021). In agreement with this, genetic *Nrf2* deletion or pharmacologic inhibition lowered systolic blood pressure in mice (Zhao et al., 2018).

In another study using chronic angiotensin II infusion, the NRF2 inducer, tert‐butylhydroquinone, amplified early (Day 0–3) angiotensin II‐induced increases in blood pressure while decreasing late (Day 8–12) blood pressures, in an NRF2‐dependent manner (Wang et al., 2018). Another inducer, cinnamaldehyde, reduced blood pressure in diabetic mice and improved vasodilation in explanted arteries (Wang et al., 2020). Similarly, the NRF2 inducer, resveratrol, reduced blood pressure in spontaneously hypertensive rats (Javkhedkar et al., 2015), and in an oxidant‐induced hypertension model, NRF2 inhibition increased blood pressure and markers of oxidative stress (Farooqui et al., 2021). Currently, it is not clear why NRF2 increases blood pressure in some studies and reduces it in others. Further study to understand how and by what mechanisms NRF2 affects blood pressure in normal and disease states is required.

## 4 ROLES IN KIDNEY DISEASE

### 4.1 Acute kidney injury (AKI)

Abundant evidence demonstrates that NRF2 activity is protective in renal ischemia–reperfusion injury (IRI). *Nrf2* knockout mice exhibited increased injury (serum creatinine and histologic tubular injury) and mortality compared to wild‐type animals (Liu et al., 2009). Conversely, mice having genetic *Keap1* modifications increasing NRF2 activity (*Keap1* hypomorphic mice and tubule‐specific *Keap1* deletion) were protected from IRI‐AKI (Nezu, Souma, et al., 2017). Lymphocyte NRF2 activity is also important, as upregulation of NRF2 specifically in CD4^+^ T cells protected against IRI (Kurzhagen et al., 2023; Noel et al., 2015), while activation in myeloid cells (macrophages and neutrophils) did not (Nezu, Souma, et al., 2017).

Pharmacologic NRF2 enhancement is also effective when administered within a therapeutic window. Sulforaphane, RTA‐408 (omaveloxolone), CDDO‐Me, and CDDO‐Im all reduced injury when administered at least 24 h prior to IRI (Han et al., 2017; Liu et al., 2014; Wu et al., 2011; Yoon et al., 2008). However, protection was not observed if CDDO‐Im treatment was delayed (starting 3 h prior to IRI or later) (Liu et al., 2014). Septic AKI is reduced by the induction of NRF2 activity prior to injury (Chen et al., 2022), and NRF2 protects against heme‐induced renal damage after intravascular hemolysis (Rubio‐Navarro et al., 2019). Interestingly, preconditioning with remote ischemia protects against AKI, and this is associated with increased NRF2 (Liu & Gong, 2015). Furthermore, prolonged intestinal ischemia causes AKI, but postconditioning with additional but brief cycles of intestinal ischemia and reperfusion similarly increased kidney NRF2 and decreased renal injury (Chen et al., 2020).

NRF2 protects against a wide variety of nephrotoxin‐induced AKI, consistent with its detoxifying and antioxidant functions. NRF2 activation reduces kidney injury caused by exposure to arsenic, cadmium, cisplatin, contrast, and methotrexate (Guerrero‐Beltran et al., 2010; Mapuskar et al., 2023; Ran et al., 2022; Salama et al., 2021; Xu et al., 2021; Younis et al., 2021), while *Nrf2* knockout mice are more susceptible to cisplatin‐induced nephrotoxic injury (Liu et al., 2009). At least some of this protection may be due to direct toxin elimination, but NRF2 may also protect against post‐exposure evolution of disease. Future studies can differentiate these effects with carefully timed activation of NRF2.

### 4.2 AKI‐to‐CKD progression

Patients with AKI have a higher risk of developing renal fibrosis and CKD (Basile et al., 2016). Studies from our laboratory and others show that higher NRF2 activity (via constitutive genetic modifications in mice) reduced fibrosis at late timepoints after IRI‐AKI (Nezu, Souma, et al., 2017; Tan et al., 2016). Nezu and colleagues further demonstrated that CDDO‐Im started 1 day after injury mitigated the development of fibrosis, but delaying administration until day 7 did not (Nezu, Souma, et al., 2017). In aged rats, basal expression of NRF2 is decreased and is associated with worse renal fibrosis after IRI, which is rescued with CDDO‐Me starting as late as 3 days after injury (Jo et al., 2023). These data reveal a post‐injury therapeutic window in which NRF2 enhancement is protective against the development of fibrosis and CKD. This is clinically relevant since most human AKI is recognized after injury has already occurred.

Since NRF2 activity reduces kidney injury, we sought to examine how its endogenous activity levels are affected by AKI. While some investigators found an increase in NRF2 activity after AKI (Leonard et al., 2006), in our hands there was prolonged suppression, which would predispose the kidney to further injury (Tan et al., 2016). In both mild and severe IRI (induced by short and long ischemia times), we found that kidney NQO1 expression was immediately reduced 1 day after injury (Bondi et al., 2022). Over the next 10 days, mice with mild injury restored normal NRF2 activity and NQO1 protein levels, recovered kidney function, and avoided renal fibrosis. Mice with severe injury exhibited permanent NRF2 suppression, nonrecovery of kidney function, and progression of renal fibrosis and CKD. Our data suggest that restoration of NRF2 activity leads to renal recovery while prolonged suppression promotes CKD. The latter may occur through the activity of hypoxia‐inducible factor‐1α (HIF‐1α), which inhibited NRF2 activity in severe injury (Bondi et al., 2022).

In one study, a regulatory role was found for GSK‐3β, a protein that facilitates NRF2 nuclear exclusion and degradation (Lu et al., 2019). In mice exhibiting recovery after folic‐acid induced AKI, GSK‐3β expression was low and NRF2 activity was high. In mice with progression to CKD, high GSK‐3β expression inhibited NRF2 activity, albeit to a level that was still higher than baseline. Inhibition of GSK‐3β with lithium chloride prevented AKI‐to‐CKD progression, even if delayed until 7 days after the injury (Lu et al., 2019).

In summary, lower NRF2 levels are associated with severe AKI. Meanwhile, early interventions to increase NRF2 activity are critical for protection and can affect the long‐term development of fibrosis and CKD. Additional studies are needed to determine how different types of AKI are affected by NRF2, and whether NRF2 activity can prevent human AKI and AKI‐to‐CKD progression.

### 4.3 Nonproteinuric CKD

NRF2 activity preserves normal kidney architecture and prevents progression of CKD. In unilateral ureteral obstruction (UUO), *Nrf2* knockout mice had increased fibrosis while *Keap1* hypomorphism reduced fibrosis (Kong et al., 2017; Tan et al., 2016). Pretreatment with CDDO‐Me protects against CKD induced by aristolochic acid (Wu et al., 2014), which is converted into electrophilic compounds that adduct to DNA (Han et al., 2019). Kidney injury in hyperuricemic nephropathy was worsened in *Nrf2* knockout mice and improved with sulforaphane (Qiao et al., 2023).

Autosomal dominant polycystic kidney disease (ADPKD) is an inherited disorder leading to cyst formation in both kidneys. ADPKD patients exhibit oxidative stress and lower NRF2 activity, while in mice, cyst growth was accelerated in *Nrf2* knockouts and reduced in animals treated with sulforaphane (Lu, Sun, et al., 2020). Tolvaptan, a vasopressin antagonist and known treatment for ADPKD, activates the NRF2 pathway in cells of the outer medulla (Fujiki et al., 2019). The FALCON trial was designed to test whether bardoxolone methyl slows ADPKD but was terminated in 2023 (https://clinicaltrials.gov/study/NCT03918447) when results of other clinical trials led the sponsor to end all of its CKD trials (see below).

### 4.4 Proteinuric CKD

Glomerular diseases such as diabetic nephropathy, IgA nephropathy, and focal segmental glomerulosclerosis (FSGS) remain important causes of kidney failure. Several clinical trials tested whether bardoxolone methyl (CDDO‐Me) is an effective treatment for these diseases. Unfortunately, there is no convincing evidence that NRF2 enhancement slows the progression of proteinuric CKD in humans.

Cancer patients treated with bardoxolone methyl exhibit increased eGFR (Hong et al., 2012), a finding that spawned several clinical trials targeting diabetic kidney disease (BEAM, BEACON, TSUBAKI, AYAME). All confirmed an early increase in eGFR (Akizawa et al., 2023; de Zeeuw et al., 2013; Nangaku et al., 2020; Pergola et al., 2011). However, the BEACON trial was terminated early due to serious adverse events including heart failure exacerbations and death, suggesting an effect on fluid retention. Bardoxolone methyl also increased albuminuria and blood pressure and caused hypomagnesemia and weight loss (de Zeeuw et al., 2013). The final trial (AYAME) examined extended treatment with bardoxolone methyl (at least 3 years) in a diabetic kidney disease population with exclusions for heart failure (Nangaku et al., 2023). In results presented recently, treatment with bardoxolone methyl did not change time to onset of ESRD (https://www.kyowakirin.com/media_center/news_releases/2023/pdf/e20230510_01.pdf; Akizawa et al., 2023).

Bardoxolone methyl was also tested in Alport syndrome patients in the CARDINAL trial. The phase 3 portion of this trial examined the effects of 2  years of exposure to bardoxolone methyl, with a 4‐week drug washout each year to address concerns about drug‐induced reversible changes in GFR. The investigators found mean improvement in eGFR after the 1‐ and 2‐year washout periods (Warady et al., 2022). However, the Food and Drug Administration (FDA) declined the new drug application, citing concerns about an insufficient washout period, increases in albuminuria and blood pressure, and weight loss in a pediatric population. Further, the FDA stated that increases in GFR appeared to be a “one‐time benefit that does not grow over time,” calling into question its overall utility in disease treatment (https://www.fda.gov/media/154630/download). In the aftermath of CARDINAL and AYAME, Reata Pharmaceuticals and its partner Kyowa Kirin terminated all of their clinical trials of bardoxolone methyl in kidney disease in 2023 (https://www.kyowakirin.com/media_center/news_releases/2023/pdf/e20230510_01.pdf).

The mechanisms for the albuminuria and GFR effects are still under study. Nonhuman primate research suggested that megalin downregulation (and lower tubular protein reabsorption) led to the increased albuminuria with bardoxolone methyl exposure (Reisman et al., 2012). An alternative explanation is that bardoxolone methyl increases intraglomerular pressure, which would hasten CKD progression, explain the increased GFR, and is the opposite effect of proven treatments such as renin angiotensin system (RAS) inhibitors (Baigent & Lennon, 2018).

Several animal studies confirm deleterious effects of NRF2 activity in proteinuric CKD. A bardoxolone methyl analog, RTA 405, worsened proteinuria and kidney injury in diabetic rats, even when combined with RAS inhibition. Concerns about purity of the drug led to studies with another analog, RTA dh404, which also worsened disease and caused kidney pseudotumors (Zoja et al., 2013). Reata Pharmaceuticals repeated this study and did not see these adverse effects, but their data still reported a large numerical increase in proteinuria in animals treated with either analog (Chin et al., 2013).

Since NRF2 inducers could have off‐target effects, we examined *Keap1* hypomorphic mice with global NRF2 hyperactivation (Rush et al., 2021). These mice had no increase in proteinuria at baseline but did exhibit increased proteinuria compared to wild‐type mice in three different glomerular injury models (Adriamycin, angiotensin II, and albumin overload). The proteinuria was accompanied by more severe glomerular injury and renal fibrosis. While *Keap1* hypomorphic mice exhibited higher blood pressures, the measured difference (~7 mmHg in mean arterial pressure) was not likely to explain the very large difference in proteinuria. Conversely, *Nrf2* knockout mice were protected from proteinuria. CDDO‐Im administration increased proteinuria in wild‐type but not the *Nrf2* knockout mice, showing dependence on *Nrf2* for the effect. (Rush et al., 2021).

In elegant studies, Zhao and colleagues showed that NRF2 can worsen proteinuria through SGLT2 (Zhao et al., 2021). While combined Akita diabetic *Nrf2* knockout mice were protected from proteinuria and renal fibrosis, the transgenic reintroduction of *Nrf2* in just the renal tubules was sufficient to restore kidney disease. The mutant mice expressed higher levels of SGLT2, and NRF2 directly promoted its gene expression in vitro (Zhao et al., 2021). Similar effects were found in the db/db mouse (Su et al., 2023). These findings point strongly to an SGLT2‐dependent mechanism for the increases in proteinuria and GFR since it is well‐known that SGLT2 inhibitors reduce GFR and proteinuria (Vallon & Verma, 2021).

However, it is acknowledged that many animal studies show that NRF2 is protective in glomerular disease. *Nrf2* knockout mice exhibited greater oxidative stress, glomerular injury and proteinuria compared to wild‐type mice in streptozotocin‐induced diabetic kidney disease (Jiang et al., 2010). Sulforaphane ameliorated diabetic nephropathy and obesity‐related glomerular disease (Cui et al., 2012; Lu, Zhang, et al., 2020; Zheng et al., 2011). Long‐term administration of RTA dh404 in 5/6 nephrectomy in rats restored NRF2 protein expression, attenuated oxidative stress, proinflammatory, and profibrotic pathways, and reduced inflammation, glomerulosclerosis, and interstitial fibrosis (Aminzadeh et al., 2014). A recent study in Akita *Nrf2* knockout mice demonstrated that lack of *Nrf2* led to worse glomerular and tubular injury, inflammation, and renal fibrosis (Liu et al., 2022).

The reasons for the divergent results from animal studies are not immediately clear, but differences in strength of NRF2 activity may play a role. In the 5/6 nephrectomy model in rats, a lower dose of RTA dh404 protected against glomerulosclerosis, interstitial fibrosis, and inflammation, while a higher dose worsened proteinuria, interstitial fibrosis, oxidative and inflammatory pathways, and paradoxically reduced NRF2 activity (Vaziri et al., 2015). Some studies, including our own unpublished observations, show that high doses of NRF2 inducers are cytotoxic or fatal (Hisamichi et al., 2018). Indeed, our studies using *Keap1* hypomorphs or CDDO‐Im did lead to strong upregulation of *Nqo1* levels, but the fact that *Nrf2* knockout mice were protected from proteinuria suggests even low NRF2 activity can be harmful (Rush et al., 2021).

NRF2 is not the only pathway with dual effects in biology. HIF‐1α and HIF‐2α, although both regulated by prolyl hydroxylases, have opposing effects on fibrosis and inflammation (Packer, 2021). The Wnt/β‐catenin pathway is another example, in which early and transient activation of the pathway is associated with renal recovery after injury, while prolonged and sustained activation leads to AKI‐to‐CKD progression (Xiao et al., 2016). Experimental design should carefully address the titratable level of NRF2 induction, method by which it is induced (pharmacologic versus genetic), and the exact timing and the cell types in which induction is achieved.

In our opinion, the current evidence does not support NRF2 enhancement in the treatment of proteinuric kidney diseases. The “benefit” of higher GFR in humans treated with bardoxolone methyl is controversial and has not been proven to provide long‐term benefit in slowing progression to ESRD. Deleterious increases in proteinuria and blood pressure have been observed in humans and recapitulated in animals when NRF2 is induced. NRF2 also appears to have poorly understood dose‐dependent effects. Future studies are needed to clarify the exact mechanisms of NRF2 effects in proteinuric diseases.

## 5 CONCLUSION

**FIGURE 2:**
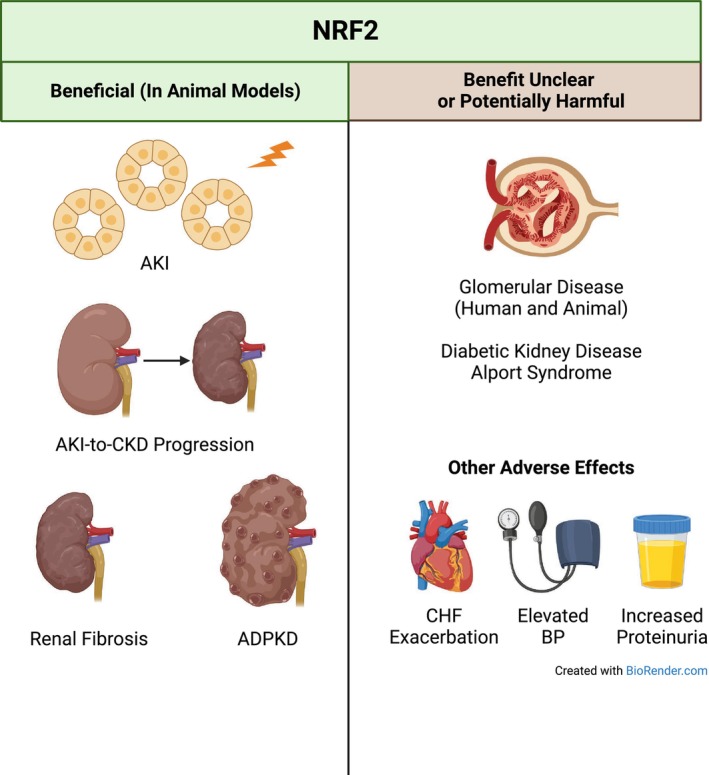
Overview of the effects of NRF2. Animal studies have shown that NRF2 activation, when properly timed, can mitigate AKI, AKI‐to‐CKD progression, renal fibrosis, and cystogenesis in autosomal dominant polycystic kidney disease (ADPKD). Conversely, in glomerular diseases such as human diabetic kidney disease and Alport syndrome, NRF2 has not been shown to have a clear effect to slow disease progression. Many animal studies also show deleterious effects in glomerular disease models. Furthermore, NRF2 appears to have negative effects on congestive heart failure (CHF) and fluid accumulation, blood pressure (BP), and even kidney disease and proteinuria.

In the kidney, NRF2 plays a role in mitigating oxidative and electrophilic stress and inflammation and in regulation of physiological processes including salt, water, and glucose handling and blood pressure. To date, the positive results from experimental models of AKI and nonproteinuric CKD demonstrate potential therapeutic value in pharmacologically enhancing NRF2 activity. Unfortunately, the use of NRF2 to treat proteinuric CKD with glomerular injury may harm patients and worsen kidney disease (Figure 2). Although the clinical trials in proteinuric CKD were appropriately terminated, study of NRF2 should not be completely abandoned. Future studies must elucidate the pros and cons of NRF2 in different disease states, at acute and chronic stages, and at different levels of activation. Potential side effects, including effects on albuminuria, magnesium, blood pressure, weight loss, and fluid retention, also require study and mitigation. This research should remain a priority, not only for the purposes of treating disease, but to better understand the impact of NRF2 activity on kidney physiology and pathology.

## ETHICS STATEMENT

Not applicable.

## Data Availability

Data sharing not applicable—no new data generated.
